# LW-AFC, a new formula derived from Liuwei Dihuang decoction, ameliorates behavioral and pathological deterioration via modulating the neuroendocrine-immune system in PrP-hAβPPswe/PS1^ΔE9^ transgenic mice

**DOI:** 10.1186/s13195-016-0226-6

**Published:** 2016-12-13

**Authors:** Jian-Hui Wang, Xi Lei, Xiao-Rui Cheng, Xiao-Rui Zhang, Gang Liu, Jun-Ping Cheng, Yi-Ran Xu, Ju Zeng, Wen-Xia Zhou, Yong-Xiang Zhang

**Affiliations:** 1 Department of Neuroimmunopharmacology, Beijing Institute of Pharmacology and Toxicology, Beijing, 100850 China; 2 State Key Laboratory of Toxicology and Medical Countermeasures, Beijing, 100850 China; 3 Guangxi Medical University, Nanning, 530021 China

**Keywords:** LW-AFC, Alzheimer’s disease, PrP-hAβPPswe/PS1^ΔE9^ transgenic mouse, cognitive behavior, Amyloid-β, Neuron loss, Neuroendocrine, Lymphocyte subset, Cytokine

## Abstract

**Background:**

Accumulating evidence implicates the neuroendocrine immunomodulation (NIM) network in the physiopathological mechanism of Alzheimer’s disease (AD). Notably, we previously revealed that the NIM network is dysregulated in the PrP-hAβPPswe/PS1^ΔE9^ (APP/PS1) transgenic mouse model of AD.

**Methods:**

After treatment with a novel Liuwei Dihuang formula (LW-AFC), mice were cognitively evaluated in behavioral experiments. Neuron loss, amyloid-β (Aβ) deposition, and Aβ level were analyzed using Nissl staining, immunofluorescence, and an AlphaLISA assay, respectively. Multiplex bead analysis, a radioimmunoassay, immunochemiluminometry, and an enzyme-linked immunosorbent assay (ELISA) were used to measure cytokine and hormone levels. Lymphocyte subsets were detected using flow cytometry. Data between two groups were compared using a Student’s *t* test. Comparison of the data from multiple groups against one group was performed using a one-way analysis of variance (ANOVA) followed by a Dunnett’s post hoc test or a two-way repeated-measures analysis of variance with a Tukey multiple comparisons test.

**Results:**

LW-AFC ameliorated the cognitive impairment observed in APP/PS1 mice, including the impairment of object recognition memory, spatial learning and memory, and active and passive avoidance. In addition, LW-AFC alleviated the neuron loss in the hippocampus, suppressed Aβ deposition in the brain, and reduced the concentration of Aβ_1–42_ in the hippocampus and plasma of APP/PS1 mice. LW-AFC treatment also significantly decreased the secretion of corticotropin-releasing hormone and gonadotropin-releasing hormone in the hypothalamus, and adrenocorticotropic hormone, luteinizing hormone, and follicle-stimulating hormone in the pituitary. Moreover, LW-AFC increased CD8^+^CD28^+^ T cells, and reduced CD4^+^CD25^+^Foxp3^+^ T cells in the spleen lymphocytes, downregulated interleukin (IL)-1β, IL-2, IL-6, IL-23, granulocyte-macrophage colony stimulating factor, and tumor necrosis factor-α and -β, and upregulated IL-4 and granulocyte colony stimulating factor in the plasma of APP/PS1 mice.

**Conclusions:**

LW-AFC ameliorated the behavioral and pathological deterioration of APP/PS1 transgenic mice via the restoration of the NIM network to a greater extent than either memantine or donepezil, which supports the use of LW-AFC as a potential agent for AD therapy.

**Electronic supplementary material:**

The online version of this article (doi:10.1186/s13195-016-0226-6) contains supplementary material, which is available to authorized users.

## Background

Alzheimer’s disease (AD) is characterized by a progressive loss of episodic memory and other cognitive functions. AD is reaching epidemic proportions, and has an enormous emotional and financial burden on individuals and society [1]. Current AD drugs target cholinergic and glutamatergic neurotransmission, thus ameliorating symptoms; however, the long-term efficacy of these drugs in clinical practice remains controversial [2–4]. Moreover, there is still no effective intervention to prevent, halt, or reverse AD. The dominant hypothesis for AD drug development is the amyloid-β (Aβ) hypothesis, and major trials of potential disease-modifying drugs have been based on the modulation of Aβ [5–11]; however, success has been elusive. These failures have led to debate about the Aβ hypothesis in the research and development of candidate drugs for AD [12].

The neuroendocrine immunomodulation (NIM) network maintains the processes of adaptation, homeostasis, and defense against hostile environmental factors [13, 14]. Changes in the NIM network have influence on the development of various diseases, ranging from immune and infectious diseases to schizophrenia, anxiety, depression, and AD [15]. Increasing evidence shows that dysregulation of the NIM network contributes to the pathogenesis of AD [16–19]. Anomalous secretions of neurotransmitters, hormones, or cytokines in a dysregulated NIM network stimulate or aggravate Aβ deposits [20, 21], tau hyperphosphorylation [22, 23], neuronal cell loss [24–26], neuroinflammation [27], and cognitive deterioration [9, 28] in AD animal models and patients.

Previously, we found that the NIM network is altered and dysfunctional in a PrP-hAβPPswe/PS1^ΔE9^ (APP/PS1) mouse model of familial AD and early-onset AD [29, 30]. In the present study, we examined the effects of long-term oral administration of a novel Liuwei Dihuang formula (LW-AFC) on cognitive impairment, Aβ deposition, and neuronal loss in APP/PS1 mice. Our results suggest that LW-AFC is a potential therapeutic agent for AD.

## Methods

### Preparation of LW-AFC and HPLC analysis

LW-AFC was prepared from an Liuwei Dihuang decoction (LW), a traditional Chinese medical prescription [31]. LW was prepared as previously described in Yang et al. [32], Zhang et al. [33, 34], Cheng et al. [35], and Kusters et al. [36]. LW-AFC is composed of a polysaccharide fraction (LWB-B), a glycoside fraction (LWD-b), and an oligosaccharide fraction (CA-30). The LW was passed through a six-layer gauze filter, and the extracted solution was centrifuged. The supernatant was concentrated and then extracted in ethanol to produce LWD. The sediment was rinsed in deionized water and concentrated into a dried polysaccharide fraction (LWB-B). The LWD ethanol elution fraction was dissolved using macroporous adsorptive resins to obtain the glycosides component (LWD-b). The water elution fraction of the LWD was dissolved using an active carbon absorption column to obtain the oligosaccharide component (CA-30). In this manner, the LW-AFC formula is composed of 20.3% of the polysaccharide component (LWB-B), 15.1% of the glycosides component (LWD-b), and 64.6% of the oligosaccharide component (CA-30) in a dry weight ratio. The LW-AFC components were analyzed using high-performance liquid chromatography (HPLC). Briefly, for the CA-30 and LWD-b mixture, chromatographic separation was obtained on a Diamond C18 column; there were 17 chromatogram peaks in the fingerprint of the CA-30 and LWD-b mixture. For LWB-B, the chromatographic separation was obtained on a NucleosilNH_2_ 100 Å column; five chromatogram peaks were observed, representing fructose, glucose, sucrose, mannotriose, and stachyose. The retention times of these peaks were 6.260 min, 6.829 min, 8.186 min, 18.305 min, and 21.506 min, respectively.

### Experimental animals

The male APP/PS1 mice and wild-type (WT) mice were obtained from Beijing HFK Bioscience Co. Ltd., via the Jackson Laboratory (Bar Harbor, ME, USA). The mice were maintained at the Beijing Institute of Pharmacology and Toxicology under standard housing conditions, i.e., room temperature at 22 ± 1 °C and humidity at 55 ± 5%, and a 12-h light/12-h dark cycle. The mice were separately given water and pellet food ad libitum (provided by the Animal Center of the Academy of Military Medical Sciences). All behavioral tests were performed between 19:00 and 6:00 (Beijing time).

**Fig. 1:**
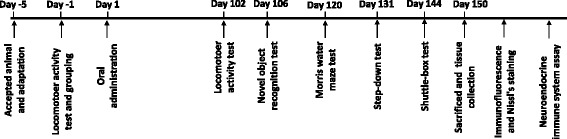
Schematic diagram of the experimental procedure

Nine-month-old male APP/PS1 mice were randomly separated into four groups. Each group contained 10–11 mice. LW-AFC was dissolved in distilled water at 160 mg/mL, memantine (Ouhe Chemical Ltd., Beijing, China) at 1 mg/mL, and donepezil (Ouhe Chemical Ltd.) at 0.1 mg/mL. The drug-treated mice group was given an intragastric administration of memantine, donepezil, or LW-AFC (0.1 mL/10 g body weight) once a day for 150 days. APP/PS1 mice as a model group and age-matched WT mice (15 males) as a control group were given an equal volume of deionized water. The mice were weighed every 3 days. Drug administration and behavioral tests were conducted according to the experimental timelines (Fig. 1). Following the behavioral experiments, the whole brain, hippocampus, cortex, hypothalamus, pituitary, spleens, and plasma of each mouse were collected for immunofluorescence, Nissl staining, soluble Aβ analysis, hormone determination, lymphocytes subsets analysis, and cytokine analysis.

### Behavioral tests

#### Locomotor activity test

The locomotor activity test was carried out according to Cheng et al. [37]. Motor tracking was performed using a video-based behavior monitoring system (Jiliang Software Technology Co. Ltd., Shanghai, China). Each mouse was placed in an aluminum-plastic panel locomotor activity chamber, and recorded for 20 min. The total distance traveled for each mouse was recorded to indicate its spontaneous motor activity.

#### Novel object recognition test

The object recognition test was performed as described by Chen et al. [38] and Bevins and Besheer [39]. The mice were familiarized with the testing environment, a black Plexiglas apparatus (30 × 30 × 30 cm), for 20 min per day for 2 days before the object recognition test. In the learning phase (day 3), the animal was placed in the apparatus with two similar black square (4 × 4 × 4 cm) Plexiglas objects, A and B, which were equidistant from the sides (5 cm) of the chamber. The animals were allowed to freely explore the chamber for 16 min. The exploration time refers to the duration that the animal spent exploring the object with their head orientated towards the object and their nose within 1 cm of the object. Then, the mice were returned to their home cage for 1 h, after which they entered a 4-min test phase, where a different object (C) replaced object A or B. The preferential index was calculated to assess the object recognition memory of mice.

#### Morris water maze test

The Morris water maze test was performed according to Vorhees and Williams [40]. During the trial, curtains with unique geometric figures were placed at all sides of the pool to avoid visual interference. The spatial learning phase consisted of four trials per day for 5 days, and one additional day (day 6) for a probe trial. In the spatial learning phase, each mouse was placed on the platform for 60 s before the first trial, and then released into the water to find the platform within 60 s. If the mouse found the platform within 60 s, it was allowed to stay there for 10 s. If not, the mouse was gently led to the platform and allowed to remain there for 10 s, the latency time being scored as 60 s. The latency time was recorded as a measure of spatial learning. For the spatial memory phase, the platform was removed, and the mouse was released into the water at a novel position and allowed to swim freely for 60 s. The dependent measure for the spatial memory was the time in the target quadrant and the number of platform crossings.

#### Step-down test

The step-down test was carried out according to Fang et al. [41], Lou et al. [42], and Shi et al. [43]. On the first day, mice were allowed to acclimatize for 2 min without the platform. During the learning trial, if the mouse stepped down from the platform (error) with all four paws it received an aversive foot electric shock (36 V, AC), and the learning course was performed for 10 min. The number of errors and the number of times that mice did not step down were scored for a 3-min period. For the testing trials (days 2–7), the procedure was repeated at the same time, and the testing time was 3 min. The number of times that the mice did not step down was recorded for a 3-min period.

#### Shuttle box test

The shuttle-box test was performed according to Cheng et al. [44]. Working memory was evaluated using a shuttle box apparatus (VT 05448, Med Associates Inc., East Fairfield, VT, USA). The training session began with 2 min of acclimatization to the chambers followed by 30 trials, and the inter-trial interval was 30 s. A tone (60 dB) and light (8 W) were presented as the conditioned stimulus, for 10 s, and followed by the unconditioned stimulus, an electrical foot shock (0.2 mA), for 5 s. If the mouse moved to the opposite chamber during the presentation of the conditioned stimulus, no electrical foot shock was presented and an active avoidance response was recorded. The shuttle-box procedure was performed for 5 consecutive days. On day 6, all of the mice were submitted to another session (no shock) to evaluate learning and memory, and the number of active avoidances was recorded.

### Biochemical and histochemical analyses

#### Immunofluorescence

Mouse brains were removed and one hemisphere was fixed via immersion in 4% paraformaldehyde in phosphate-buffered saline (PBS) (pH 7.4) at 4 °C overnight, and then fixed in 10% buffered formalin, and paraffin embedded. Serial 5-μm thick sections were prepared, deparaffinized, hydrated, rinsed with PBS, and pretreated with 0.01 M citric acid for 15 min for antigen retrieval, and then with the blocking solution (2% fetal bovine serum in PBS) for 30 min. Subsequently, sections were incubated with mouse anti-β-amyloid (clone: 6E10, 1:100; Biolegend, San Diego, CA, USA) overnight at 4 °C. After rinsing, the sections were incubated with goat anti-mouse IgG HRP (1:1000; ZSGB-Bio, Beijing, China) for 2 h at room temperature, then incubated with Opal520 working solution (PerkinElmer, Waltham, MA, USA) for 10 min, and mounted with DAPI-containing medium. The tissue sections were photographed, and the images were digitized with a fluorescence lifetime imaging microscope (Vectra 2, PerkinElmer-Caliper LS, Waltham, MA, USA). The area of Aβ deposits in each slice section was quantified using Image Pro Plus 6.0 software.

#### Nissl staining

The sections were stained using 0.5% cresyl violet acetate (Beyotime, Beijing China). Stained sections were scanned using a transmission electron microscope (H-7650, Hitachi, Tokyo, Japan). The integrated optical density (IOD) of Nissl bodies in the CA1 and CA3 regions was quantified using the Image Pro Plus 6.0 software.

#### Soluble Aβ analysis

The Aβ AlphaLISA assay was carried out according to Cheng et al. [45] and Tesseur et al. [46]. The hippocampus and cortex from one brain hemisphere of each mouse was sequentially extracted. The extracted tissue was separately homogenized using an ultrasonic disintegrator in 50 mmol/L Tris-HCl, pH 8.0, and 5 mol/L guanidine hydrochloride. After 3 h at room temperature, the suspension was diluted in Dulbecco’s phosphate-buffered saline (0.03% Tween-20, 5% fetal bovine serum, PBS, pH 7.4) and complete proteinase inhibitor cocktail (Roche, Indianapolis, IN, USA), centrifuged at 16,000 g for 20 min at 4 °C, and the supernatants containing the soluble fraction were used for measuring soluble Aβ. Plasma was prepared from the obtained supernatant by centrifuging the blood plus 4% EDTA-Na_2_ at 3000 g for 15 min at 4 °C. The Aβ_1–40_ and Aβ_1–42_ content in the hippocampus, cortex and plasma were determined using the AlphaLISA technique and the AlphaLISA® human amyloid beta 1–40 (high specificity) (AL275C, PerkinElmer, Waltham, MA, USA) and AlphaLISA® human amyloid beta 1–42 (high specificity) (AL276C, PerkinElmer) Kits according to the manufacturers’ instructions.

#### Radioimmunoassay of hypothalamic and hypophyseal hormones

The hypothalamuses and pituitaries were weighed and boiled in 1 mL of saline for 5 min. Peptides were extracted by homogenizing the hypothalamuses and pituitaries in 0.5 mL of 1 mol/L glacial acetic acid followed by centrifuging the mixture at 3000 rpm for 30 min. Supernatants were stored at –20 °C. Concentrations of adrenocorticotropic hormone (ACTH), luteinizing hormone (LH), follicle-stimulating hormone (FSH), corticotropin releasing hormone (CRH), and gonadotropin-releasing hormone (GnRH) in the supernatants were determined with a ^125^I-ACTH RIA kit (North Institute of Biological Technology, Beijing, China), a ^125^I-LH RIA kit (North Institute of Biological Technology), a ^125^I-FSH RIA kit (North Institute of Biological Technology), a ^125^I-CRH RIA kit (Department of Neurobiology of the Second Military Medical University, Shanghai, China ), and a ^125^I-GnRH RIA kit (Department of Neurobiology of the Second Military Medical University), respectively.

#### Enzyme-linked immunosorbent assay

The corticosterone (CORT) level in the plasma of the mice was measured using a precoated corticosterone enzyme-linked immunosorbent assay (ELISA) kit (EC3001-1, ASSAYPRO, Charles, MO, USA) according to the manufacturer’s instruction. The absorbance was measured at 450 nm with a reference wavelength of 570 nm using Enspire™ multilabel reader 2300 (Perkin Elmer, Turku, Finland).

#### Immunochemiluminescence assay

The level of testosterone (T) in the plasma of the mice was measured using an Access Immunoassay System (Beckman Coulter, Brea, CA, USA), access testosterone (33560, Beckman Coulter), and access testosterone calibrators (33565, Beckman Coulter). The entire measurement was automatically processed according to the scheduled program.

#### Flow cytometric analysis

Mouse spleen cells were harvested and divided into three parts. The first portion of the spleen cells was treated with 100 μL of 20 μg/mL FITC anti-mouse CD3 antibody (BioLegend, San Diego, CA, USA), 100 μL of 12.5 μg/mL PerCP anti-mouse CD4 antibody (BioLegend), 100 μL of 25 μg/mL APC anti-mouse CD25 antibody (BioLegend), and 100 μL of 20 μg/mL PE anti-mouse Foxp3 antibody (BioLegend) at 25 °C for 30 min, washed, and then incubated with 100 μL of 10 μg/mL FITC-conjugated goat anti-rat IgG (BioLegend) at 25 °C for 30 min in the dark. The second portion of the spleen cells was treated with 100 μL of 20 μg/mL FITC anti-mouse CD3 antibody (BioLegend), 100 μL of 12.5 μg/mL APC anti-mouse CD8 antibody (BioLegend), and 100 μL of 12.5 μg/mL PE anti-mouse CD28 antibody (BioLegend) using the same protocol as above. The third portion of spleen cells was treated with 100 μL of 20 μg/mL FITC anti-mouse CD19 antibody (BioLegend) and 100 μL of 50 μg/mL PE anti-mouse CD80 antibody (BioLegend) using the same protocol as above. After incubation, the cells were washed and resuspended in 0.5 mL of PBS/2% paraformaldehyde, and then quantified using flow cytometry (BD Calibur™, San Jose, CA, USA).

#### Multiplex bead analysis

Plasma samples of the mice were analyzed using multiplex bead analysis. The manufacturer’s instructions were followed to measure interleukin (IL)-1β, IL-2, IL-5, IL-17, IL-6, IL-4, IL-10, granulocyte-macrophage colony stimulating factor (GM-CSF), granulocyte colony stimulating factor (G-CSF), interferon (IFN)-γ, tumor necrosis factor (TNF)-α, monocyte chemotactic protein (MCP)-1, regulated upon activation normal T cell expressed and secreted factor (RANTES), eotaxin, macrophage inflammatory protein (MIP)-1β, IL-23, and TNF-β (Millipore Corp., Billerica, MA, USA). The samples were analyzed using Luminex 200™ (Luminex, Austin, TX, USA). The levels of IL-1β, IL-2, IL-5, IL-17, IL-6, IL-4, IL-10, GM-CSF, G-CSF, IFN-γ, TNF-α, MCP-1, RANTES, eotaxin, and MIP-1β were detected using a multiplex map kit (MCYTOMAG-70 K, Millipore). IL-23 and TNF-β were detected using another multiplex map kit (MGAMMAG-300 K, Millipore).

### Statistical analysis

All data are expressed as the mean ± SEM. GraphPad Prism 6.0 (GraphPad Software, Inc., La Jolla, CA, USA) was used to plot and analyze the data. Data between two groups were compared using a Student’s *t* test. Comparison of the data from multiple groups against one group was performed using a one-way analysis of variance (ANOVA) followed by a Dunnett’s post hoc test or a two-way repeated-measures analysis of variance with a Tukey multiple comparisons test. *P* < 0.05 was considered statistically significant.

## Results

### LW-AFC improves the cognitive impairment of APP/PS1 mice

**Fig. 2:**
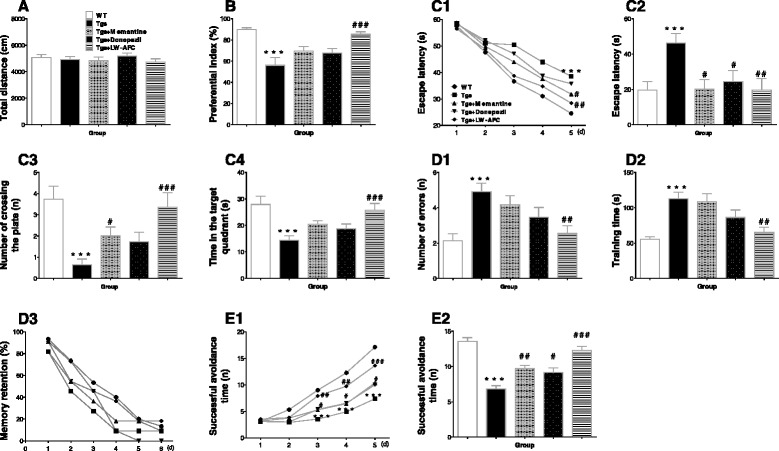
The effect of LW-AFC on the cognitive deterioration of APP/PS1 mice. Shown are: **a** the total distance in a spontaneous locomotor activity test; **b** the preferential index (time on novel object C/( time on novel object C + time on sample object A) × 100% ) in a novel object recognition test; the escape latency of **c1** learning task and **c2** probe trial, **c3** number of times that the mice crossed the removed platform, and **c4** swimming time in the target quadrant in the Morris water maze test; The **d1** number of errors, **d2** training time, and **d3** memory retention in the step-down test; and the successful avoidance times during **e1** training and **e2** testing phases in the shuttle-box test. The values are mean ± SEM; *n* = 11–15. *P < 0.05, ***P < 0.001, versus the wild-type (*WT*) mouse group by unpaired Student's *t* test; ^#^ *P* < 0.05, ^##^ *P* < 0.01, ^###^ *P* < 0.001, versus the APP/PS1 (*Tgs*) mouse group by one-way ANOVA analysis followed by Dunnett’s post hoc test and two-way repeated-measures analysis of variance with Tukey multiple comparisons test

The locomotor activity test evaluated the spontaneous motor activity of APP/PS1 mice; no significant difference was observed between the treatment groups (Fig. 2a). This result indicates that the spontaneous locomotor activity of mice did not influence the results of the other behavioral experiments.

The novel object recognition test evaluated the object recognition memory of mice. LW-AFC treatment in APP/PS1 mice significantly decreased the preferential index (Fig. 2b), indicating that the object recognition memory deficit of APP/PS1 mice was ameliorated after LW-AFC treatment; this effect was superior to that of memantine or donepezil.

The Morris water maze test evaluated the spatial learning and memory of APP/PS1 mice. For the learning task, APP/PS1 mice had longer escape latencies than WT mice on the final test day, and the latencies of the LW-AFC- or memantine-treated APP/PS1 mice were significantly longer than those of the mice in the non-treated group (Fig. 2c1). These results indicate that both LW-AFC and memantine ameliorated the spatial learning impairment of APP/PS1 mice. For the probe trial, the escape latency was longer (Fig. 2c2), the number of plate crossings decreased (Fig. 2c3), and the time in the target quadrant was shorter (Fig. 2c4), but swimming speed was not significantly different (data not shown) in APP/PS1 mice compared with WT mice. The escape latencies decreased and the number of plate crossings increased in LW-AFC- or memantine-treated mice, while the time in the target quadrant was elevated only in mice treated with LW-AFC. These results indicate that LW-AFC and memantine administration significantly improved the spatial learning and memory deficits of APP/PS1 mice.

The step-down test evaluated passive avoidance in APP/PS1 mice. Compared with the WT mice, the number of errors and training time in APP/PS1 mice significantly increased (Fig. 2d1 and d2), and the memory retention of APP/PS1 mice tended to decrease (Fig. 2d3). LW-AFC administration significantly decreased the number of errors and training time. These results indicate that LW-AFC improved the passive avoidance impairment of APP/PS1 mice; this effect was greater than that observed for either memantine or donepezil.

The shuttle-box test evaluated active avoidance in APP/PS1 mice. Significantly fewer successful avoidance times were observed for APP/PS1 mice than for WT mice after the third day of the training phase, but significantly increased after treatment with either LW-AFC, memantine, or donepezil after the third day (Fig. 2e1). Significantly shorter successful avoidance times were observed for APP/PS1 mice than for WT mice in the testing phase, but increased after LW-AFC, memantine, or donepezil administration (Fig. 2e2). These results indicate that the deteriorated active avoidance response of APP/PS1 mice was ameliorated after LW-AFC, memantine, or donepezil administration.

### LW-AFC treatment decreases neuronal loss in the hippocampus of APP/PS1 mice

**Fig. 3:**
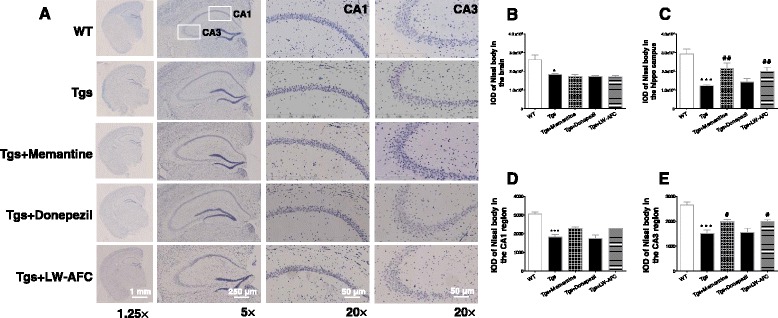
The LW-AFC treatment alleviated neuron loss in the hippocampus of APP/PS1 mice. **a** Representative Nissl staining images showing Nissl bodies in the CA1 and CA3 regions of the hippocampus in wild-type (*WT* ) and APP/PS1 (*Tgs*) mice. Quantification of Nissl bodies in the brain (**b**), hippocampus (**c**), CA1 (**d**), and CA3 (**e**) regions of hippocampus in WT and Tgs mice by Image Pro Plus 6.0 software. The values are mean ± SEM; *n* = 11–15. *P < 0.05, ***P < 0.001, versus the WT mouse group by unpaired Student's *t* test; ^#^ *P* < 0.05, ^##^ *P* < 0.01, versus the Tgs mouse group by one-way ANOVA analysis followed by Dunnett’s post hoc test. *IOD* integrated optical density

Nissl staining revealed typical neuropathological changes in the CA1 and CA3 region of the hippocampus in APP/PS1 mice compared to WT mice, including neuron loss and nucleus shrinkage or disappearance (Fig. 3a). Furthermore, significantly lower Nissl body numbers were observed in the whole brain, hippocampus, and CA1 and CA3 regions of APP/PS1 mice than in those regions in WT mice (Fig. 3b–e). LW-AFC and memantine treatment significantly decreased these neuropathological changes and increased the density of healthy neurons in the hippocampus and CA3 region of APP/PS1 mice (Fig. 3c and e). These findings indicate that LW-AFC and memantine protected against neuronal loss in the hippocampus of APP/PS1 mice.

### LW-AFC treatment alleviates Aβ deposition in the brain of APP/PS1 mice

**Fig. 4:**
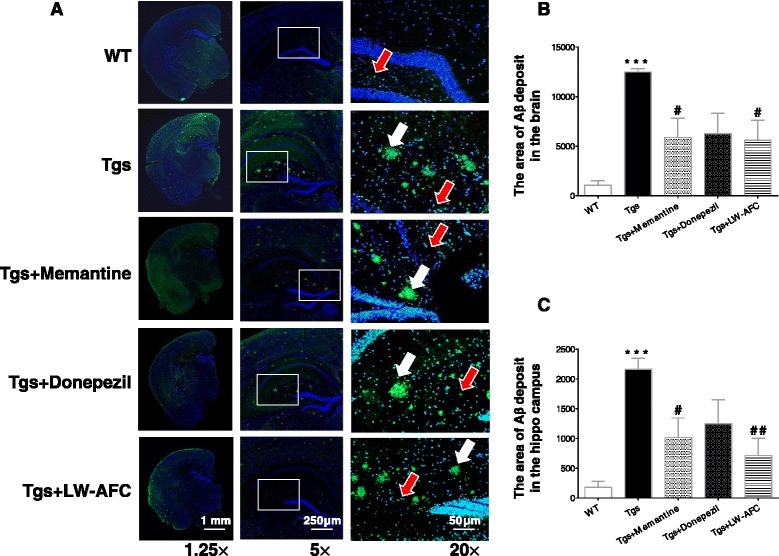
Suppressive effects of LW-AFC on Aβ deposits in the hippocampus of APP/PS1 mice. **a** Representative immunofluorescence staining images showing amyloid-β (*Aβ*) deposits (*green* and indicated by *white arrows*) in the hippocampus and brain of wild-type (*WT*) and APP/PS1 (*Tgs*) mice. Quantification of Aβ deposits in the brain (**b**) and hippocampus (**c**) of WT and Tgs mice by Image Pro Plus 6.0 software. *Red arrows* indicate false positive result of Aβ deposits. The values are mean ± SEM; *n* = 11–15. **P < 0.01, ***P < 0.001, versus the WT mice by unpaired Student's *t* test; ^#^ *P* < 0.05, ^##^ *P* < 0.01, versus the Tgs mice by one-way ANOVA analysis followed by Dunnett’s post hoc test

Aβ deposition in the brain is a typical pathological sign of AD in patients and animal models. Our results show that APP/PS1 mice developed a significant number of Aβ plaques in the brain at 14 months, while Aβ plaques were not observed in the WT mice (Fig. 4a). LW-AFC- or memantine-treated mice had a significantly smaller area of Aβ deposits in the whole brain and hippocampus, while donepezil had a less prominent influence on Aβ plaque formation in APP/PS1 mice (Fig. 4b and c). These results indicate that the Aβ deposition in the brain of APP/PS1 mice was alleviated after LW-AFC or memantine administration.

### LW-AFC treatment decreases Aβ_1–40_ and Aβ_1–42_ levels in the brain and blood of APP/PS1 mice

**Fig. 5:**
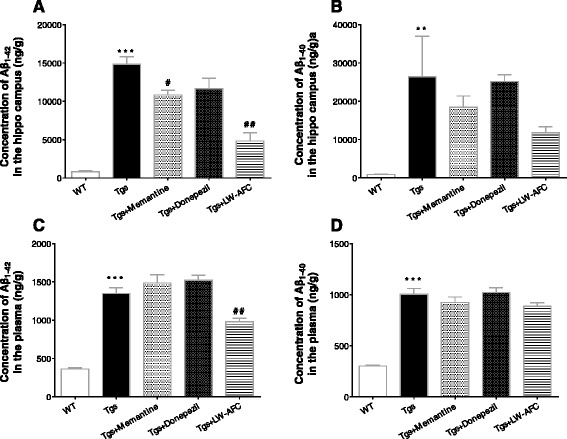
The effect of LW-AFC on the concentration of amyloid-β (*Aβ*) in the hippocampus and plasma of APP/PS1 mice. The concentration of Aβ_1–42_ and Aβ_1–40_ in the brain (**a** and **b**) and plasma (**c** and **d**) of wild-type (*WT*) and APP/PS1 (*Tgs*) mice was investigated by AlphaLISA assay . The values are mean ± SEM; *n* = 11–15. **P < 0.01, ***P < 0.001, versus the WT mice by unpaired Student's *t* test; ^#^ *P* < 0.05, ^##^ *P* < 0.01, versus the Tgs mice by one-way ANOVA analysis followed by Dunnett’s post hoc test

The results of the AlphaLISA assay showed that the concentrations of Aβ_1–42_ and Aβ_1–40_ in the hippocampus and plasma of APP/PS1 mice were significantly higher than that of WT mice (Fig. 5). LW-AFC or memantine treatment led to significantly lower Aβ_1–42_ levels in the hippocampus of APP/PS1 mice than in those of WT mice, and Aβ_1–42_ levels in the plasma were decreased only after LW-AFC administration. These findings indicate that LW-AFC downregulates Aβ_1–42_ levels in the brain and blood of APP/PS1 mice, while similar effects for memantine were observed only in the brain.

### LW-AFC restores the NIM network in APP/PS1 mice

**Fig. 6:**
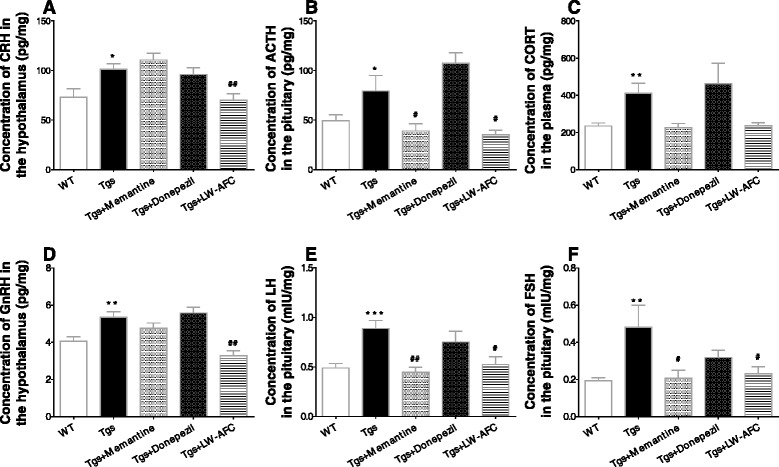
The effect of LW-AFC on the hormone secretion of the HPA and HPG axes in APP/PS1 mice. The hormones of the HPA axis including **a** corticotropin releasing hormone (*CRH*) in the hypothalamus, **b** adrenocorticotropic hormone (*ACTH*) in the pituitary, and **c** corticosterone (*CORT*) in the plasma. The HPG axis including **d** gonadotropin-releasing hormone (*GnRH*) in the hypothalamus, **e** luteinizing hormone (*LH*), and **f** follicle-stimulating hormone (*FSH*) in the pituitary. The values are mean ± SEM; *n* = 9–11. *P < 0.05, **P < 0.01, ***P < 0.001, versus the wild-type (*WT*) mouse group by unpaired Student's *t* test; ^#^ *P* < 0.05, ^##^ *P* < 0.01, versus the APP/PS1 (*Tgs*) mouse group by one-way ANOVA analysis followed by Dunnett’s post hoc test

To further investigate whether LW-AFC affected the hypothalamic–pituitary–adrenal (HPA) axis and the hypothalamic–pituitary–gonadal (HPG) axis in APP/PS1 mice, the concentration of GnRH and CRH in the hypothalamus, and ACTH, FSH, and LH in the pituitary, were measured using a radioimmunoassay. The concentration of T and CORT in the plasma was measured using a chemiluminescence assay and ELISA, respectively. The results show that, within the HPA axis, the concentration of CRH, ACTH, and CORT were significantly higher in APP/PS1 mice than in WT mice (Fig. 6a–c). LW-AFC significantly decreased the CRH level (Fig. 6a), while both LW-AFC and memantine reduced ACTH (Fig. 6b). Within the HPG axis, the concentration of GnRH, FSH, and LH increased in APP/PS1 mice (Fig. 6d–f), but T (data not shown) was not significantly different between the APP/PS1 and WT mice. LW-AFC significantly decreased the concentration of GnRH (Fig. 6d), while both LW-AFC and memantine reduced FSH and LH levels (Fig. 6e and f) in APP/PS1 mice. These data indicate that both LW-AFC and memantine had an ameliorative effect on the endocrine system in APP/PS1 mice, especially the HPA and HPG axes.

A Pearson correlation analysis was performed to identify altered endocrine hormone expression after LW-AFC treatment that was associated with cognitive impairment, neuron loss, and Aβ deposition in APP/PS1 mice. The results show that among the endocrine hormones with altered expression after LW-AFC treatment, CRH levels were correlated with neuron loss and Aβ deposits in APP/PS1 mice. ACTH levels were correlated with object recognition memory, spatial memory, and an active avoidance response. GnRH levels were correlated with object recognition memory, an active avoidance response, and neuron loss. LH levels were correlated with an active avoidance response and neuron loss. FSH levels were correlated with object recognition memory, a passive avoidance response, an active avoidance response, and neuron loss in APP/PS1 mice (see Additional file 1: Table S1).

### LW-AFC modulates the impairment of lymphocyte subsets in APP/PS1 mice

**Fig. 7:**
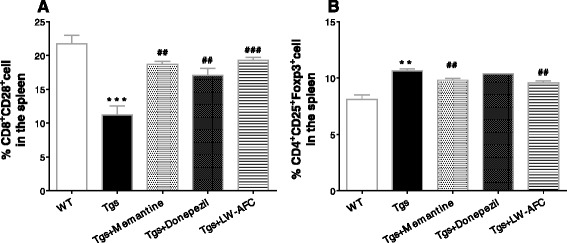
The effect of LW-AFC on the subsets of spleen lymphocytes in APP/PS1 mice. Flow cytometric analysis of CD8^+^CD28^+^ T cells (**a**) and CD4^+^CD25^+^Foxp3^+^ T cells (**b**) in the spleen supernatant of mice. The values are mean ± SEM; *n* = 3. **P < 0.01, ***P < 0.001, versus the wild-type (*WT*) mouse group by unpaired Student's *t* test; ^##^ *P* < 0.01, ^###^ *P* < 0.001, versus the APP/PS1 (*Tgs*) mouse group by one-way ANOVA analysis followed by Dunnett’s post hoc test

To investigate the effect of LW-AFC on the expression of lymphocyte subsets in APP/PS1 mice, the expression of CD3^+^CD4^+^ T cells, CD3^+^CD8^+^ T cells, CD8^+^CD28^+^ T cells, CD3^+^CD25^+^Foxp3^+^ T cells, CD19^+^ B cells, and CD19^+^CD80^+^ B cells were detected using flow cytometry. Significantly fewer CD8^+^CD28^+^ T cells (Fig. 7a) and significantly more CD3^+^CD25^+^Foxp3^+^ T cells (Fig. 7b) were observed in APP/PS1 mice than in WT mice. Expression of the other lymphocyte subsets in APP/PS1 mice (data not shown) was not significantly different. In APP/PS1 mice, the expression of CD8^+^CD28^+^ T cells increased after memantine, donepezil, or LW-AFC treatment (Fig. 7a), while the expression of CD4^+^CD25^+^Foxp3^+^ T cells decreased after memantine or LW-AFC treatment (Fig. 7b). These results indicate that memantine or LW-AFC treatment partially restored normal lymphocyte expression in APP/PS1 mice.

### LW-AFC modulates abnormal cytokine production in APP/PS1 mice

**Fig. 8:**
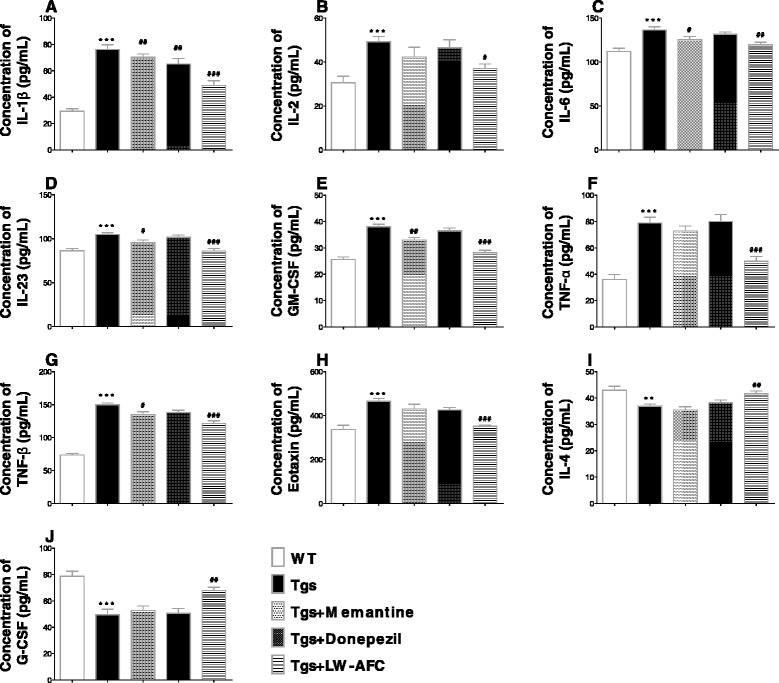
The effect of LW-AFC on the cytokines in the plasma of APP/PS1 mice. Concentrations (pg/mL) of **a** interleukin-1β (*IL-1β*), **b** interleukin-2 (*IL-2*), **c** interleukin-6 (*IL-6*), **d** interleukin-23 (*IL-23*), **e** granulocyte-macrophage colony stimulating factor (*GM-CSF*), **f** tumor necrosis factor α (*TNF-α*), **g** tumor necrosis factor β (*TNF-β*), **h** eotaxin, **i** interleukin-4 (*IL-4*), and **j** granulocyte colony stimulating factor (*G-CSF*) in the blood plasma of wild-type (*WT*) and APP/PS1 (*Tgs*) mice were detected using Luminex® X-MAP® technology. The values are mean ± SEM; *n* = 9–15. **P < 0.01, ***P < 0.001, versus WT mouse group by unpaired Student's *t* test; ^#^ *P* < 0.05, ^##^ *P* < 0.01, ^###^ *P* < 0.001, versus Tgs mouse group by one-way ANOVA analysis followed by Dunnett’s post hoc test

Multiplex bead analysis was used to detect the concentration of pro-inflammatory cytokines (IL-1β, IL-2, IL-6, IL-23, IL-17, GM-CSF, IFN-γ, TNF-α, TNF-β, RANTES, eotaxin, MCP-1, and MIP-1β) and anti-inflammatory cytokines (IL-4, IL-5, IL-10, and G-CSF) in the blood plasma of APP/PS1 mice. Increases in the levels of IL-1β, IL-2, IL-6, IL-23, GM-CSF, TNF-α, TNF-β, and the chemotactic factor eotaxin, and decreases in the levels of IL-4 and G-CSF in the blood plasma were observed in APP/PS1 mice compared to WT mice (Fig. 8). The secretion of other cytokines in APP/PS1 mice (data not shown) was not significantly different. Memantine treatment significantly decreased the production of IL-1β, IL-6, IL-23, GM-CSF, and TNF-β, while treatment with only donepezil decreased IL-1β production in the blood plasma of APP/PS1 mice. The levels of IL-1β, IL-2, IL-6, IL-23, GM-CSF, TNF-α, TNF-β, and eotaxin were decreased; IL-4 and G-CSF were elevated after LW-AFC administration (Fig. 8). These results indicate that cytokine secretion in APP/PS1 mice was abnormal and that administration of LW-AFC and memantine restored this aberrant immune function in APP/PS1 mice.

The results of a Pearson correlation analysis showed that among the cytokines with altered expression after LW-AFC administration, the levels of IL-1β, GM-CSF, TNF-α, TNF-β, and eotaxin were correlated with object recognition memory, spatial learning and memory, passive avoidance response, active avoidance response, neuron loss, and Aβ deposits in APP/PS1 mice. IL-2 levels were correlated with object recognition memory, spatial learning and memory, an active avoidance response, and neuron loss. IL-6 and G-CSF levels were correlated with object recognition memory, spatial memory, a passive avoidance response, an active avoidance response, neuron loss, and Aβ deposits. IL-23 levels were correlated with object recognition memory, spatial memory, a passive avoidance response, and an active avoidance response. IL-4 levels were correlated with object recognition memory, spatial memory, a passive avoidance response, an active avoidance response, and Aβ deposits (see Additional file 1: Table S1 and Additional file 2: Figure S1).

## Discussion

In AD patients, cognitive impairments and psychological symptoms are associated with an early dysfunction of the HPA [47] and HPG axes [48–51]. However, few studies have investigated neuroendocrine function in APP/PS1 mice or analogous models, although one study reported that plasma CORT levels were increased in 8-month-old male APP/PS1 mice [52]. In addition, the neuroendocrine system is disturbed in Aβ knockin- or injection-induced mouse models [23, 53–56]. Several lines of evidence also indicate that hormones in the neuroendocrine system regulate pathogenic Aβ accumulation in AD animal models and patients [20, 22, 57–60]. In the present study, the HPA and HPG axes were significantly disturbed in APP/PS1 mice as compared with those of age-matched WT mice. These disturbances might contribute to the acceleration of Aβ pathogenesis, neuron loss, and cognitive impairment. Our results show that long-term treatment with LW-AFC restored the neuroendocrine system of APP/PS1 mice, while memantine had little impact on the function of the HPA and HPG axes.

The immune system has a fundamental role in the development and progression of AD. AD patients show altered lymphocyte expression that includes CD4^+^ and CD8^+^ T cells [61–66], CD8^+^CD28^+^ T cells [65], CD4^+^CD25^+^Foxp3^+^ T cells [67], and B cells [66, 68, 69]. AD patients have increased levels of CD8^+^CD28^+^ T cells and reduced levels of cytotoxic CD28^–^ cells, which leads to T helper cell unresponsiveness [65]. Nevertheless, we found lower levels of CD8^+^CD28^+^ T cells in APP/PS1 mice than in WT mice. This discrepancy might be due to the overexpression of a Swedish variant of the gene encoding hAPP along with the overexpression of a mutant PS1 gene in APP/PS1 mice [70], but not a multifactorial inducer in AD patients. CD4^+^CD25^+^Foxp3^+^ T cells were also elevated in AD patients [64, 65, 71], which may be related to AD progression, as patients with a mild cognitive impairment (MCI) show lower CD4^+^CD25^+^Foxp3^+^ T-cell activity than AD patients [67]. Our results show that LW-AFC or memantine administration ameliorated this change in CD4^+^CD25^+^Foxp3^+^ T cells in APP/PS1 mice.

Cytokines play a critical role in brain inflammation in AD, and are involved in complex cognitive processes [72]. An aberrant cytokine concentration is found in AD patients [73], and contributes to the impairment of learning and memory [72]. IL-1β, IL-2, and IL-6 modulate synaptic transmission and plasticity in the hippocampus [72], inhibit long-term potentiation [74, 75], affect various forms of hippocampal-dependent memory [76], and impair planning [77]. The upregulation of IL-23 contributes to age-associated brain dysfunction, the modulation of Aβ, and neuronal loss [24, 78]. High levels of GM-CSF in the plasma of AD transgenic mice correlate with the expansion of regulatory T cells that suppress the effector T-cell response to Aβ_1–42_ [79]. Moreover, increasing the peripheral eotaxin concentration in young mice inhibited adult neurogenesis and learning and memory [80]. AD model mice treated with G-CSF show a significant reversal of cognitive deficits, and decreased Aβ deposition and soluble Aβ levels in the periphery and hippocampus [21]. The contribution of IL-23 to AD pathogenesis is not fully understood [81]. Previous studies demonstrated that the cytokine network in APP/PS1 mice is indiscriminate as compared to that in WT mice [82–86]. In the present study, we measured 17 cytokines in the plasma of APP/PS1 mice and found that long-term administration of LW-AFC ameliorated the secretion of 10 of these cytokines. The association of many of these cytokines (including eotaxin, G-CSF, IL-2, IL-23, and TNF-β) with AD-like learning and memory deficits in APP/PS1 mice is a novel observation.

Our study shows that, in APP/PS1 mice, the concentrations of TNF-β and IL-1β in the blood are correlated with object recognition memory, spatial learning and memory, a passive or active avoidance response, neuron loss, and Aβ deposits, and could be regulated by LW-AFC treatment. Under normal immunological conditions, TNF is essential for learning and memory [87]. The cognitive dysfunction induced in mice after overexpression of TNF might be due to decreased nerve growth factor (NGF) levels [88] and/or the modulation of synaptic plasticity [89–91], impaired long-term potentiation (LTP), and neurodegeneration [92]. The TNF receptor is crucial for Aβ-mediated cell death [93, 94]. Elevated cerebrospinal fluid (CSF) levels of soluble TNF receptors is observed in patients with MCI and in AD patients [95], and chronic administration of thalidomide, a well-known TNF-α inhibitor, reduced Aβ load, plaque formation, and BACE1 levels and activity in APP23 transgenic mice [96]. Our results show that chronic administration of LW-AFC decreased the elevated levels of TNF-α and TNF-β in the blood of APP/PS1 mice. Some studies report that overexpressing IL-1β leads to an increase in the microglia and astrocytes surrounding Aβ plaques in AD patients and animal models [97, 98]. Moreover, patients with MCI and AD exhibited a significant increase in peripheral IL-1β levels compared to controls [99]. Sustained hippocampal IL-1β overexpression exacerbated tau phosphorylation and tangle formation via aberrant activation of p38 mitogen-activated protein kinase and glycogen synthase kinase 3 [100, 101], and impaired long-term contextual and spatial memory [102]. Some agents can alleviate AD symptoms via inhibiting IL-1β release, such as nimodipine [103], *Gossypium herbaceam* L. extracts [104], linalool [105], melatonin [106], and anti-IL-1R blocking antibody [100]. The present study shows that LW-AFC treatment reverses the decrease in IL-1β levels in the blood of APP/PS1 mice.

## Conclusions

**Fig. 9:**
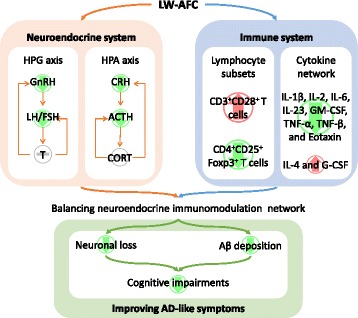
LW-AFC ameliorated behavioral and pathological deterioration via modulation of the neuroendocrine immune system;  represents a decrease,  represents an increase, and represents no influence

Taken together, the results of the present investigation show that LW-AFC improves cognitive impairment, and reduces Aβ deposits and neuron loss in APP/PS1 mice via modulation of the neuroendocrine immune system (Fig. 9). These findings support the use of LW-AFC as a potential therapeutic agent for AD.

## Additional files

Additional file 1: Table S1. Correlation between endocrine hormone/cytokines and cognitive performance/pathology index of APP/PS1 mice. (PDF 495 kb)
Additional file 2: Figure S1. Correlation between endocrine hormones /cytokines and cognitive performance/pathology index of APP/PS1 mice. (PDF 420 kb)

## Abbreviations

ACTH: Adrenocorticotropic hormone
AD: Alzheimer’s disease
ANOVA: Analysis of variance
APP/PS1: PrP-hAβPPswe/PS1^ΔE9^
Aβ: Amyloid-β
CORT: Corticosterone
CRH: Corticotropin releasing hormone
ELISA: Enzyme-linked immunosorbent assay
FSH: Follicle-stimulating hormone
G-CSF: Granulocyte colony stimulating factor
GM-CSF: Granulocyte-macrophage colony stimulating factor
GnRH: Gonadotropin-releasing hormone
HPA: Hypothalamic–pituitary–adrenal
HPG: Hypothalamic–pituitary–gonadal
IFN: Interferon
IL: Interleukin
LH: Luteinizing hormone
LW: Liuwei Dihuang decoction
MCI: Mild cognitive impairment
MCP: Monocyte chemotactic protein
MIP: Macrophage inflammatory protein
NIM: Neuroendocrine immunomodulation
PBS: Phosphate-buffered saline
RANTES: Regulated upon activation normal T cell expressed and secreted factor
T: Testosterone
TNF: Tumor necrosis factor
WT: Wild-type

## References

[1] Huang Y, Mucke L (2012). Alzheimer mechanisms and therapeutic strategies. Cell.

[2] Courtney C, Farrell D, Gray R, Hills R, Lynch L, Sellwood E, Edwards S, Hardyman W, Raftery J, Crome P (2004). Long-term donepezil treatment in 565 patients with Alzheimer’s disease (AD2000): randomised double-blind trial. Lancet.

[3] Blacker D (2006). Neither vitamin E nor donepezil delays progression from amnestic mild cognitive impairment to Alzheimer’s disease in the long term. Evid Based Ment Health.

[4] Dysken MW, Sano M, Asthana S, Vertrees JE, Pallaki M, Llorente M, Love S, Schellenberg GD, McCarten JR, Malphurs J (2014). Effect of vitamin E and memantine on functional decline in Alzheimer disease: the TEAM-AD VA cooperative randomized trial. Jama.

[5] Gilman S, Koller M, Black RS, Jenkins L, Griffith SG, Fox NC, Eisner L, Kirby L, Rovira MB, Forette F (2005). Clinical effects of Abeta immunization (AN1792) in patients with AD in an interrupted trial. Neurology.

[6] Coric V, van Dyck CH, Salloway S, Andreasen N, Brody M, Richter RW, Soininen H, Thein S, Shiovitz T, Pilcher G (2012). Safety and tolerability of the gamma-secretase inhibitor avagacestat in a phase 2 study of mild to moderate Alzheimer disease. Arch Neurol.

[7] Bapineuzumab phase 3: target engagement, but no benefit [http://www.alzforum.org/news/conference-coverage/european-federation-neurological-societies]. Accessed 11 Sept 2012.

[8] Sampson EL, Jenagaratnam L, McShane R (2014). Metal protein attenuating compounds for the treatment of Alzheimer’s dementia. Cochrane Database Syst Rev..

[9] Green RC, Schneider LS, Amato DA, Beelen AP, Wilcock G, Swabb EA, Zavitz KH (2009). Effect of tarenflurbil on cognitive decline and activities of daily living in patients with mild Alzheimer disease: a randomized controlled trial. Jama.

[10] Doody RS, Raman R, Farlow M, Iwatsubo T, Vellas B, Joffe S, Kieburtz K, He F, Sun X, Thomas RG (2013). A phase 3 trial of semagacestat for treatment of Alzheimer’s disease. N Engl J Med.

[11] Doody RS, Thomas RG, Martin F, Takeshi I, Bruno V, Steven J, Karl K, Rema R, Xiaoying S, Aisen PS (2014). Phase 3 trials of solanezumab for mild-to-moderate Alzheimer’s disease. N Engl J Med.

[12] Smith AD (2010). Why are drug trials in Alzheimer’s disease failing?. Lancet.

[13] Masek K, Slansky J, Petrovicky P, Hadden JW (2003). Neuroendocrine immune interactions in health and disease. Int Immunopharmacol.

[14] Bellavance MA, Rivest S (2012). The neuroendocrine control of the innate immune system in health and brain diseases. Immunol Rev.

[15] Bilbo SD, Klein SL (2012). Special issue: the neuroendocrine-immune axis in health and disease. Horm Behav.

[16] Reese LC, Taglialatela G (2010). Neuroimmunomodulation by calcineurin in aging and Alzheimer’s disease. Aging Dis.

[17] Maccioni RB, Rojo LE, Fernandez JA, Kuljis RO (2009). The role of neuroimmunomodulation in Alzheimer’s disease. Ann N Y Acad Sci..

[18] Gimenez-Llort L, Arranz L, Mate I, De la Fuente M (2008). Gender-specific neuroimmunoendocrine aging in a triple-transgenic 3xTg-AD mouse model for Alzheimer’s disease and its relation with longevity. Neuroimmunomodulation.

[19] Morales I, Farias G, Maccioni RB (2010). Neuroimmunomodulation in the pathogenesis of Alzheimer’s disease. Neuroimmunomodulation.

[20] Verdile G, Laws SM, Henley D, Ames D, Bush AI, Ellis KA, Faux NG, Gupta VB, Li QX, Masters CL (2014). Associations between gonadotropins, testosterone and beta amyloid in men at risk of Alzheimer’s disease. Mol Psychiatry.

[21] Sanchez-Ramos J, Song S, Sava V, Catlow B, Lin X, Mori T, Cao C, Arendash GW (2009). Granulocyte colony stimulating factor decreases brain amyloid burden and reverses cognitive impairment in Alzheimer’s mice. Neuroscience.

[22] Green KN, Billings LM, Roozendaal B, McGaugh JL, LaFerla FM (2006). Glucocorticoids increase amyloid-beta and tau pathology in a mouse model of Alzheimer’s disease. J Neurosci.

[23] Brureau A, Zussy C, Delair B, Ogier C, Ixart G, Maurice T, Givalois L (2013). Deregulation of hypothalamic-pituitary-adrenal axis functions in an Alzheimer’s disease rat model. Neurobiol Aging.

[24] Tan M-S, Yu J-T, Jiang T, Zhu X-C, Guan H-S, Tan L (2014). IL12/23 p40 inhibition ameliorates Alzheimer’s disease-associated neuropathology and spatial memory in SAMP8 mice. J Alzheimers Dis.

[25] Kang L, Li S, Xing Z, Li J, Su Y, Fan P, Wang L, Cui H (2014). Dihydrotestosterone treatment delays the conversion from mild cognitive impairment to Alzheimer’s disease in SAMP8 mice. Horm Behav.

[26] Tripathy D, Thirumangalakudi L, Grammas P (2010). RANTES upregulation in the Alzheimer’s disease brain: a possible neuroprotective role. Neurobiol Aging.

[27] Jiang H, Liu CX, Feng JB, Wang P, Zhao CP, Xie ZH, Wang Y, Xu SL, Zheng CY, Bi JZ (2010). Granulocyte colony-stimulating factor attenuates chronic neuroinflammation in the brain of amyloid precursor protein transgenic mice: an Alzheimer’s disease mouse model. J Int Med Res.

[28] Bowen RL, Perry G, Xiong C, Smith MA, Atwood CS (2015). A clinical study of lupron depot in the treatment of women with Alzheimer’s disease: preservation of cognitive function in patients taking an acetylcholinesterase inhibitor and treated with high dose lupron over 48 weeks. J Alzheimers Dis.

[29] Cheng XR, Zhou WX, Zhang YX (2014). The behavioral, pathological and therapeutic features of the senescence-accelerated mouse prone 8 strain as an Alzheimer’s disease animal model. Ageing Res Rev..

[30] Wang JH, Cheng XR, Zhang XR, Wang TX, Xu WJ, Li F, Liu F, Cheng JP, Bo XC, Wang SQ (2016). Neuroendocrine immunomodulation network dysfunction in SAMP8 mice and PrP-hAβPPswe/PS1ΔE9 mice: potential mechanism underlying cognitive impairment. Oncotarget.

[31] Wang J, Cheng X, Zhang X, Cheng J, Xu Y, Zeng J, Zhou W, Zhang Y. The anti-aging effects of LW-AFC via correcting immune dysfunctions in senescence accelerated mouse resistant 1 (SAMR1) strain. Oncotarget. 2016;7(19):26949–965. 10.18632/oncotarget.8877 PMC5053624 27105505

[32] Yang Y, Cheng XR, Zhang GR, Zhou WX, Zhang YX (2012). Autocrine motility factor receptor is involved in the process of learning and memory in the central nervous system. Behav Brain Res.

[33] Zhang GR, Cheng XR, Zhou WX, Zhang YX (2009). Age-related expression of calcium/calmodulin-dependent protein kinase II A in the hippocampus and cerebral cortex of senescence accelerated mouse prone/8 mice is modulated by anti-Alzheimer’s disease drugs. Neuroscience.

[34] Zhang GR, Cheng XR, Zhou WX, Zhang YX (2008). Age-related expression of STUB1 in senescence-accelerated mice and its response to anti-Alzheimer’s disease traditional Chinese medicine. Neurosci Lett.

[35] Cheng XR, Zhou WX, Zhang YX (2007). The effects of Liuwei Dihuang decoction on the gene expression in the hippocampus of senescence-accelerated mouse. Fitoterapia.

[36] Kusters MAA, Verstegen RHJ, de Vries E (2011). Down syndrome: is it really characterized by precocious immunosenescence?. Aging Dis.

[37] Huang Y, Hu Z, Liu G, Zhou W, Zhang Y. Cytokines induced by long-term potentiation (LTP) recording: a potential explanation for the lack of correspondence between learning/memory performance and LTP. Neuroscience. 2013;231(3):432–443. 10.1016/j.neuroscience.2012.11.010 23201254

[38] Chen Y, Rex CS, Rice CJ, Dube CM, Gall CM, Lynch G, Baram TZ (2010). Correlated memory defects and hippocampal dendritic spine loss after acute stress involve corticotropin-releasing hormone signaling. Proc Natl Acad Sci U S A.

[39] Bevins RA, Besheer J (2006). Object recognition in rats and mice: a one-trial non-matching-to-sample learning task to study ‘recognition memory’. Nat Protoc.

[40] Vorhees CV, Williams MT (2006). Morris water maze: procedures for assessing spatial and related forms of learning and memory. Nat Protoc.

[41] Fang F, Lue LF, Yan S, Xu H, Luddy JS, Chen D, Walker DG, Stern DM, Yan S, Schmidt AM (2010). RAGE-dependent signaling in microglia contributes to neuroinflammation, Abeta accumulation, and impaired learning/memory in a mouse model of Alzheimer’s disease. FASEB J.

[42] Lou G, Zhang Q, Xiao F, Xiang Q, Su Z, Zhang L, Yang P, Yang Y, Zheng Q, Huang Y (2012). Intranasal administration of TAT-haFGF attenuates disease progression in a mouse model of Alzheimer’s disease. Neuroscience..

[43] Shi YQ, Huang TW, Chen LM, Pan XD, Zhang J, Zhu YG, Chen XC (2010). Ginsenoside Rg1 attenuates amyloid-beta content, regulates PKA/CREB activity, and improves cognitive performance in SAMP8 mice. J Alzheimers Dis.

[44] Cheng XR, Yang Y, Zhou WX, Zhang YX (2011). Expression of VGLUTs contributes to degeneration and acquisition of learning and memory. Neurobiol Learn Mem.

[45] Cheng X, Zhou Y, Gu W, Wu J, Nie A, Cheng J, Zhou J, Zhou W, Zhang Y (2013). The selective BACE1 inhibitor VIa reduces amyloid-beta production in cell and mouse models of Alzheimer’s disease. J Alzheimers Dis.

[46] Tesseur I, Pimenova AA, Lo AC, Ciesielska M, Lichtenthaler SF, De Maeyer JH, Schuurkes JA, D’Hooge R, De Strooper B (2013). Chronic 5-HT4 receptor activation decreases Abeta production and deposition in hAPP/PS1 mice. Neurobiol Aging.

[47] Swanwick GR, Kirby M, Bruce I, Buggy F, Coen RF, Coakley D, Lawlor BA (1998). Hypothalamic-pituitary-adrenal axis dysfunction in Alzheimer’s disease: lack of association between longitudinal and cross-sectional findings. Am J Psychiatry.

[48] McGonigal G, Thomas B, McQuade C, Starr JM, MacLennan WJ, Whalley LJ (1993). Epidemiology of Alzheimer’s presenile dementia in Scotland, 1974–88. BMJ.

[49] Brookmeyer R, Gray S, Kawas C (1998). Projections of Alzheimer’s disease in the United States and the public health impact of delaying disease onset. Am J Public Health.

[50] Fratiglioni L, Viitanen M, von Strauss E, Tontodonati V, Herlitz A, Winblad B (1997). Very old women at highest risk of dementia and Alzheimer’s disease: incidence data from the Kungsholmen Project, Stockholm. Neurology..

[51] Smith MA, Perry G, Atwood CS, Bowen RL (2003). Estrogen replacement and risk of Alzheimer disease. Jama.

[52] Sierksma AS, van den Hove DLA, Rutten K, Chouliaras L, Rostamian S, Steinbusch HWM, Prickaerts J. Chronic phosphodiesterase type 2 inhibition improves spatial memory and alters synaptic density in the hippocampus in the APPswe/PS1dE9 mouse model of Alzheimer’s disease. Neuropharmacology. 2013;64(1):124–136. 10.1016/j.neuropharm.2012.06.048 22771768

[53] Carroll JC, Iba M, Bangasser DA, Valentino RJ, James MJ, Brunden KR, Lee VM, Trojanowski JQ (2011). Chronic stress exacerbates tau pathology, neurodegeneration, and cognitive performance through a corticotropin-releasing factor receptor-dependent mechanism in a transgenic mouse model of tauopathy. J Neurosci.

[54] Dong H, Yuede CM, Yoo HS, Martin MV, Deal C, Mace AG, Csernansky JG (2008). Corticosterone and related receptor expression are associated with increased beta-amyloid plaques in isolated Tg2576 mice. Neuroscience.

[55] Horgan J, Miguel-Hidalgo JJ, Thrasher M, Bissette G (2007). Longitudinal brain corticotropin releasing factor and somatostatin in a transgenic mouse (TG2576) model of Alzheimer’s disease. J Alzheimers Dis.

[56] Guo Q, Zheng H, Justice NJ (2012). Central CRF system perturbation in an Alzheimer’s disease knockin mouse model. Neurobiol Aging.

[57] Kiyota T, Okuyama S, Swan RJ, Jacobsen MT, Gendelman HE, Ikezu T (2010). CNS expression of anti-inflammatory cytokine interleukin-4 attenuates Alzheimer’s disease-like pathogenesis in APP + PS1 bigenic mice. FASEB J.

[58] Webber KM, Perry G, Smith MA, Casadesus G (2007). The contribution of luteinizing hormone to Alzheimer disease pathogenesis. Clin Med Res.

[59] Rosario ER, Carroll JC, Pike CJ (2012). Evaluation of the effects of testosterone and luteinizing hormone on regulation of beta-amyloid in male 3xTg-AD mice. Brain Res..

[60] Bayatti N, Behl C (2005). The neuroprotective actions of corticotropin releasing hormone. Ageing Res Rev.

[61] Jozwik A, Landowski J, Bidzan L, Fuelop T, Bryl E, Witkowski JM. Beta-amyloid peptides enhance the proliferative response of activated CD4(+)CD28(+) lymphocytes from Alzheimer disease patients and from healthy elderly. PloS One. 2011;7(3):1250–1267. 10.1371/journal.pone.0033276 PMC3299766 22428008

[62] Larbi A, Pawelec G, Witkowski JM, Schipper HM, Derhovanessian E, Goldeck D, Fulop T (2009). Dramatic shifts in circulating CD4 but not CD8 T cell subsets in mild Alzheimer’s disease. J Alzheimers Dis.

[63] Lombardi VRM, Garcia M, Rey L, Cacabelos R (1999). Characterization of cytokine production, screening of lymphocyte subset patterns and in vitro apoptosis in healthy and Alzheimer’s disease (AD) individuals. J Neuroimmunol.

[64] Pellicano M, Larbi A, Goldeck D, Colonna-Romano G, Buffa S, Bulati M, Rubino G, Iemolo F, Candore G, Caruso C (2012). Immune profiling of Alzheimer patients. J Neuroimmunol.

[65] Speciale L, Calabrese E, Saresella M, Tinelli C, Mariani C, Sanvito L, Longhi R, Ferrante P (2007). Lymphocyte subset patterns and cytokine production in Alzheimer’s disease patients. Neurobiol Aging.

[66] Lueg G, Gross CC, Lohmann H, Johnen A, Kemmling A, Deppe M, Groger J, Minnerup J, Wiendl H, Meuth SG (2015). Clinical relevance of specific T-cell activation in the blood and cerebrospinal fluid of patients with mild Alzheimer’s disease. Neurobiol Aging.

[67] Saresella M, Calabrese E, Marventano I, Piancone F, Gatti A, Calvo MG, Nemni R, Clerici M (2010). PD1 negative and PD1 positive CD4 + T regulatory cells in mild cognitive impairment and Alzheimer’s disease. J Alzheimers Dis.

[68] Schindowski K, Kratzsch T, Peters J, Steiner B, Leutner S, Touchet N, Maurer K, Czech C, Pradier L, Frolich L (2003). Impact of aging. Neruomol Med.

[69] Richartz-Salzburger E, Batra A, Stransky E, Laske C, Koehler N, Bartels M, Buchkremer G, Schott K (2007). Altered lymphocyte distribution in Alzheimer’s disease. J Psychiatr Res.

[70] Radde R, Bolmont T, Kaeser SA, Coomaraswamy J, Lindau D, Stoltze L, Calhoun ME, Jaggi F, Wolburg H, Gengler S (2006). Abeta42-driven cerebral amyloidosis in transgenic mice reveals early and robust pathology. EMBO Rep.

[71] May JE, Pemberton RM, Hart JP, McLeod J, Wilcock G, Doran O (2013). Use of whole blood for analysis of disease-associated biomarkers. Anal Biochem.

[72] McAfoose J, Baune BT (2009). Evidence for a cytokine model of cognitive function. Neurosci Biobehav Rev.

[73] Valentine AD, Meyers CA (2005). Neurobehavioral effects of interferon therapy. Curr Psychiatr Rep.

[74] Murray CA, Lynch MA (1998). Evidence that increased hippocampal expression of the cytokine interleukin-1 beta is a common trigger for age- and stress-induced impairments in long-term potentiation. J Neurosci.

[75] Cunningham AJ, Murray CA, O’Neill LA, Lynch MA, O’Connor JJ (1996). Interleukin-1 beta (IL-1 beta) and tumour necrosis factor (TNF) inhibit long-term potentiation in the rat dentate gyrus in vitro. Neurosci Lett.

[76] Brennan FX, Beck KD, Servatius RJ (2003). Low doses of interleukin-1beta improve the leverpress avoidance performance of Sprague-Dawley rats. Neurobiol Learn Mem.

[77] Capuron L, Ravaud A, Dantzer R (2001). Timing and specificity of the cognitive changes induced by interleukin-2 and interferon-alpha treatments in cancer patients. Psychosom Med.

[78] Vom Berg J, Prokop S, Miller KR, Obst J, Kalin RE, Lopategui-Cabezas I, Wegner A, Mair F, Schipke CG, Peters O (2012). Inhibition of IL-12/IL-23 signaling reduces Alzheimer’s disease-like pathology and cognitive decline. Nat Med.

[79] Cao C, Arendash GW, Dickson A, Mamcarz MB, Lin X, Ethell DW (2009). Abeta-specific Th2 cells provide cognitive and pathological benefits to Alzheimer’s mice without infiltrating the CNS. Neurobiol Dis.

[80] Villeda SA, Luo J, Mosher KI, Zou B, Britschgi M, Bieri G, Stan TM, Fainberg N, Ding Z, Eggel A (2011). The ageing systemic milieu negatively regulates neurogenesis and cognitive function. Nature.

[81] Swardfager W, Lanctot K, Rothenburg L, Wong A, Cappell J, Herrmann N (2010). A meta-analysis of cytokines in Alzheimer’s disease. Biol Psychiatry.

[82] Gallagher JJ, Minogue AM, Lynch MA (2013). Impaired performance of female APP/PS1 mice in the Morris water maze is coupled with increased A beta accumulation and microglial activation. Neurodegener Dis.

[83] Barrett JP, Minogue AM, Jones RS, Ribeiro C, Kelly RJ, Lynch MA (2015). Bone marrow-derived macrophages from A beta PP/PS1 mice are sensitized to the effects of inflammatory stimuli. J Alzheimers Dis.

[84] Danielyan L, Klein R, Hanson LR, Buadze M, Schwab M, Gleiter CH, Frey WH (2010). Protective effects of intranasal losartan in the APP/PS1 transgenic mouse model of Alzheimer disease. Rejuvenation Res.

[85] Lv C, Wang L, Liu X, Yan S, Yan SS, Wang Y, Zhang W (2015). Multi-faced neuroprotective effects of geniposide depending on the RAGE-mediated signaling in an Alzheimer mouse model. Neuropharmacology..

[86] Schmole AC, Lundt R, Ternes S, Albayram O, Ulas T, Schultze JL, Bano D, Nicotera P, Alferink J, Zimmer A (2015). Cannabinoid receptor 2 deficiency results in reduced neuroinflammation in an Alzheimer’s disease mouse model. Neurobiol Aging.

[87] Baune BT, Wiede F, Braun A, Golledge J, Arolt V, Koerner H (2008). Cognitive dysfunction in mice deficient for TNF- and its receptors. Am J Med Genet B Neuropsychiatr Genet.

[88] Fiore M, Angelucci F, Alleva E, Branchi I, Probert L, Aloe L (2000). Learning performances, brain NGF distribution and NPY levels in transgenic mice expressing TNF-alpha. Behav Brain Res.

[89] Small DH (2008). Network dysfunction in Alzheimer’s disease: does synaptic scaling drive disease progression?. Trends Mol Med.

[90] Tobinick EL, Gross H (2008). Rapid improvement in verbal fluency and aphasia following perispinal etanercept in Alzheimer’s disease. BMC Neurol..

[91] Tobinick EL, Gross H (2008). Rapid cognitive improvement in Alzheimer’s disease following perispinal etanercept administration. J Neuroinflammation..

[92] Capsoni S, Cattaneo A (2006). On the molecular basis linking nerve growth factor (NGF) to Alzheimer’s disease. Cell Mol Neurobiol.

[93] Ping H, Zhenyu Z, Kristina L, Lilian B, Wendy L, Cynthia L, Matthias S, Rena L, Yong S (2007). Deletion of tumor necrosis factor death receptor inhibits amyloid beta generation and prevents learning and memory deficits in Alzheimer’s mice. J Exp Med.

[94] Jiang HY (2014). Genetic deletion of TNFRII gene enhances the Alzheimer-like pathology in an APP transgenic mouse model via reduction of phosphorylated IκBα. Hum Mol Genet.

[95] Hong J, Hampel H, Prvulovic D, Wallin A, Blennow K, Li R, Yong S (2011). Elevated CSF levels of TACE activity and soluble TNF receptors in subjects with mild cognitive impairment and patients with Alzheimer’s disease. Mol Neurodegener.

[96] He P, Cheng X, Staufenbiel M, Li R, Shen Y (2013). Long-term treatment of thalidomide ameliorates amyloid-like pathology through inhibition of β-secretase in a mouse model of Alzheimer’s disease. Plos One.

[97] Boutajangout A, Wisniewski T (2013). The innate immune system in Alzheimer’s disease. Int J Cell Biol.

[98] Hunter JM, Kwan J, Malek-Ahmadi M, Maarouf CL, Kokjohn TA, Belden C, Sabbagh MN, Beach TG, Roher AE (2012). Morphological and pathological evolution of the brain microcirculation in aging and Alzheimer’s disease. Plos One.

[99] Forlenza OV, Diniz BS, Talib LL, Mendonça VA, Ojopi EB, Gattaz WF, Teixeira AL (2009). Increased serum IL-1β level in Alzheimer’s disease and mild cognitive impairment. Dement Geriatr Cogn Disord.

[100] Kitazawa M, Cheng D, Tsukamoto MR, Koike MA, Wes PD, Vasilevko V, Cribbs DH, Laferla FM (2011). Blocking IL-1 signaling rescues cognition, attenuates tau pathology, and restores neuronal β-catenin pathway function in an Alzheimer's disease model. J Immunol.

[101] Sheng J, Zhu S, Jones RA, Griffin W, Mrak R (2000). Interleukin-1 promotes expression and phosphorylation of neurofilament and tau proteins in vivo. Exp Neurol.

[102] Hein AM, Stasko MR, Matousek SB, Scott-Mckean JJ, Maier SF, Olschowka JA, Costa ACS, O’Banion MK (2009). Sustained hippocampal IL-1β overexpression impairs contextual and spatial memory in transgenic mice. Brain Behav Immun.

[103] Sanz JM, Chiozzi P, Colaianna M, Zotti M, Ferrari D, Trabace L, Zuliani G, Virgilio FD (2012). Nimodipine inhibits IL-1β release stimulated by amyloid β from microglia. Br J Pharmacol.

[104] Ji C, Song C, Aisa HA, Yang N, Liu YY, Li Q, Zhu HB, Zuo PP (2012). Gossypium herbaceam L. extracts ameliorate disequilibrium of IL-1RA/IL-1β ratio to attenuate inflammatory process induced by amyloid β in rats. Curr Alzheimer Res.

[105] Angélica Maria SG, Edison O, Gloria Patricia CG (2015). Linalool reverses neuropathological and behavioral impairments in old triple transgenic Alzheimer’s mice. Neuropharmacology..

[106] Shen Y, Zhang G, Liu L, Xu S (2007). Suppressive effects of melatonin on amyloid-β-induced glial activation in rat hippocampus. Arch Med Res.

