# Supplementary material for: LW-AFC, a new formula derived from Liuwei Dihuang decoction, ameliorates behavioral and pathological deterioration via modulating the neuroendocrine-immune system in PrP-hAβPPswe/PS1ΔE9 transgenic mice
Source: Alzheimers Res Ther. 2016 Dec 13;8:57. doi: 10.1186/s13195-016-0226-6 (PMC5154149; doi:10.1186/s13195-016-0226-6)
Supplement: Additional file 1: Table S1. — Correlation between endocrine hormone/cytokines and cognitive performance/pathology index of APP/PS1 mice. (PDF 495 kb) [file 13195_2016_226_MOESM1_ESM.pdf]

Supplemental table 1 Correlation between endocrine hormone/cytokines and cognitive performance/pathology index of APP/PS1 mice

Endocrine hormone/ cytokines		Novel object recognition test	Morris water maze test				Step down test		Shuttle box test	Immunofluorescence		Nissl staining			
		PI	Escape latency of learning task	Escape latency of probe trial	Number of crossing the plate	Time in the target quadrant	Number of errors	Training time	Successful avoidance time	Aβ in brain	Aβ in hippocampus	Nissl bodis in brain	Nissl bodies in hippocampus	Nissl bodies in CA1 region	Nissl bodies in CA3 region
CRH	*R* square	0.031	0.007684	7.57E-05	0.006952	0.003694	0.05516	0.09479	0.06535	0.1897	0.2221	0.4921	0.4192	0.5258	0.4829
	P	0.2365	0.5579	0.9537	0.5774	0.6849	0.112	0.0533	0.0829	0.0378	0.0232	0.0004	0.0015	0.0002	0.0005
ACTH	*R* square	0.1025	0.05063	0.05437	0.09253	0.1247	0.05455	0.01737	0.1214	0.05796	0.1018	3.18E-05	0.05069	0.1284	0.1009
	P	0.0172	0.0986	0.0867	0.024	0.0082	0.0861	0.3718	0.0091	0.2685	0.1378	0.9807	0.3265	0.1107	0.1606
CORT	*R* square	0.061	0.009866	0.000358	0.00537	0.008529	0.1929	0.09948	0.05155	0.1725	0.2825	0.04563	0.2745	0.2115	0.2492
	P	0.0746	0.4792	0.893	0.602	0.5107	0.001	0.0308	0.1021	0.0487	0.0091	0.3525	0.0148	0.036	0.0212
GnRH	*R* square	0.1127	0.000597	0.02948	0.06312	0.04185	0.03684	0.04956	0.1743	0.03984	0.1151	0.1012	0.1905	0.2248	0.196
	P	0.0094	0.8543	0.1935	0.0549	0.1201	0.1453	0.1127	0.001	0.3612	0.1132	0.16	0.0479	0.0299	0.0445
LH	*R* square	0.03704	0.01545	0.02882	0.05176	0.05483	0.03732	0.02325	0.1843	0.1391	0.05079	0.000374	0.08844	0.2145	0.2399
	P	0.1553	0.3614	0.211	0.0917	0.0824	0.1537	0.2905	0.001	0.0797	0.3012	0.9337	0.1905	0.0345	0.0242
FSH	*R* square	0.06904	0.008756	0.0461	0.02206	0.0277	0.02863	0.08466	0.1535	0.1171	0.04978	0.000731	0.07691	0.224	0.236
	P	0.0463	0.4848	0.1055	0.2659	0.2118	0.2041	0.0364	0.0024	0.1101	0.3062	0.9074	0.2236	0.0302	0.0256
T	*R* square	0.03077	0.1029	0.03527	0.03977	0.008013	0.006416	0.01706	0.07075	0.03287	0.08864	0.03331	0.2206	0.0682	0.103
	P	0.1839	0.0132	0.1543	0.1299	0.5002	0.5465	0.356	0.0417	0.4077	0.1677	0.4285	0.0317	0.2528	0.1561
IL-1β	*R* square	0.3987	0.2428	0.0474	0.1772	0.1776	0.2815	0.3063	0.4539	0.2979	0.3646	0.3052	0.5296	0.5479	0.6198

	P	< 0.0001	< 0.0001	0.01165	0.0009	0.0009	< 0.0001	< 0.0001	< 0.0001	0.0071	0.0023	0.0094	0.0002	0.0001	< 0.0001
IL-2	*R* square	0.134	0.09559	0.4159	0.1212	0.06371	0.05921	0.05494	0.2552	0.03045	0.07128	0.1088	0.246	0.2126	0.2206
	P	0.0044	0.0172	0.0976	0.0069	0.0538	0.0633	0.0944	< 0.0001	0.4259	0.2181	0.1443	0.0222	0.0354	0.0317
IL-6	*R* square	0.2483	0.03841	0.09568	0.1829	0.1329	0.1844	0.1719	0.2203	0.2918	0.3352	0.09409	0.2182	0.281	0.3799
	P	< 0.0001	0.1368	0.0171	0.0007	0.0045	0.0007	0.0022	0.0002	0.0078	0.0038	0.1762	0.0328	0.0134	0.0029
IL-17	*R* square	0.06136	0.06663	0.01024	0.1073	0.02774	0.09225	0.09341	0.1351	4.94E-05	3.32E-05	0.0278	0.008256	0.1186	0.03951
	P	0.0585	0.0484	0.4456	0.0113	0.2074	0.0194	0.0276	0.0042	0.9746	0.9792	0.4701	0.6953	0.1263	0.3877
IL-23	*R* square	0.2041	0.04467	0.0159	0.09602	0.1532	0.06524	0.07489	0.2386	0.07613	0.1162	0.01152	0.06142	0.07301	0.1003
	P	0.0003	0.1081	0.3413	0.0169	0.0022	0.0509	0.0496	< 0.0001	0.2025	0.1115	0.6433	0.2787	0.2362	0.1619
GM-CSF	*R* square	0.2602	0.1231	0.03276	0.1549	0.1959	0.3078	0.4278	0.3996	0.2242	0.3224	0.06425	0.216	0.2106	0.2004
	P	< 0.0001	0.0064	0.1701	0.002	0.0004	< 0.0001	< 0.0001	< 0.0001	0.0225	0.0047	0.2676	0.0338	0.0364	0.0419
INF-γ	*R* square	0.1242	0.007414	0.02365	0.0607	0.04459	0.05326	0.07318	0.05753	0.2367	0.2647	0.09308	0.2958	0.368	0.2792
	P	0.0062	0.5167	0.2449	0.06	0.1084	0.0786	0.0524	0.0673	0.0186	0.012	0.1787	0.0108	0.0036	0.0138
TNF-α	*R* square	0.3592	0.1412	0.08964	0.1689	0.2071	0.1876	0.2959	0.3783	0.1891	0.2855	0.1801	0.4166	0.2824	0.3511
	P	< 0.0001	0.0034	0.0212	0.0012	0.0003	0.0006	< 0.0001	< 0.0001	0.0381	0.0086	0.0552	0.0016	0.0132	0.0046
TNF-β	*R* square	0.2471	0.2607	0.09146	0.21	0.2041	0.2568	0.2731	0.4251	0.2818	0.3687	0.4406	0.5592	0.598	0.5647
	P	< 0.0001	< 0.0001	0.0199	0.0003	0.0003	< 0.0001	< 0.0001	< 0.0001	0.0092	0.0021	0.001	< 0.0001	< 0.0001	< 0.0001
MCP-1	*R* square	0.000427	0.009826	8.37E-05	3.78E-08	0.0255	0.03927	4.32E-06	0.004932	0.008878	0.01674	0.07518	0.02452	0.003547	0.009985
	P	0.8765	0.4551	0.9452	0.9988	0.227	0.1325	0.9883	0.5971	0.6689	0.5563	0.2291	0.4978	0.7976	0.6665
MIP-1β	*R* square	0.03699	0.004091	0.00053	0.000318	0.04999	0.006839	0.001915	0.003526	0.008917	0.03721	0.01176	0.04212	0.01765	0.00035
	P	0.1445	0.6303	0.8626	0.8934	0.0887	0.5335	0.758	0.6551	0.6682	0.3778	0.6399	0.3722	0.5659	0.9359
RANTES	*R* square	0.003348	0.08904	0.03061	0.02316	0.003115	0.001741	0.000433	0.000211	0.002278	0.004425	0.03726	0.02278	0.009746	7.03E-06

	P	0.6633	0.0217	0.1851	0.2499	0.6746	0.7537	0.8837	0.9131	0.8288	0.763	0.4018	0.5137	0.6703	0.9909
Eotaxin	*R* square	0.2393	0.1137	0.06415	0.1448	0.1471	0.2788	0.2425	0.416	0.2175	0.2508	0.1018	0.1327	0.3547	0.436
	P	< 0.0001	0.0096	0.0551	0.0032	0.003	< 0.0001	0.0002	< 0.0001	0.0249	0.0149	0.1587	0.1045	0.0044	0.0011
IL-4	*R* square	0.1564	0.06538	0.0173	0.1517	0.1312	0.05906	0.1761	0.1994	0.1094	0.23	0.02422	0.1382	3.1E-06	0.00514
	P	0.0019	0.0506	0.3208	0.0023	0.0048	0.0637	0.002	0.0004	0.1232	0.0206	0.5006	0.0971	0.994	0.7575
IL-5	*R* square	0.000229	0.02918	0.008307	0.007322	0.001086	0.0125	0.000807	0.002861	0.002572	0.04581	0.1103	0.01311	0.006234	0.001634
	P	0.9102	0.1998	0.4962	0.523	0.806	0.4034	0.8432	0.6901	0.8183	0.3268	0.1414	0.6212	0.7337	0.8619
G-CSF	*R* square	0.2194	0.1706	0.08999	0.2332	0.1153	0.08748	0.2516	0.3158	0.1215	0.1182	0.3939	0.07081	0.2662	0.1254
	P	0.0002	0.0011	0.021	0.0001	0.0085	0.0229	0.0002	< 0.0001	0.1032	0.1083	0.0023	0.2436	0.0166	0.1153

(Blue) The endocrine hormone or cytokine could be ameliorated by LW-AFC administration.

(Yellow) The endocrine hormone or cytokine was significantly correlated with cognitive performance or pathology index.
