# Supplementary material for: LW-AFC, a new formula derived from Liuwei Dihuang decoction, ameliorates behavioral and pathological deterioration via modulating the neuroendocrine-immune system in PrP-hAβPPswe/PS1ΔE9 transgenic mice
Source: Alzheimers Res Ther. 2016 Dec 13;8:57. doi: 10.1186/s13195-016-0226-6 (PMC5154149; doi:10.1186/s13195-016-0226-6)
Supplement: Additional file 2: Figure S1. — Correlation between endocrine hormones /cytokines and cognitive performance/pathology index of APP/PS1 mice. (PDF 420 kb) [file 13195_2016_226_MOESM2_ESM.pdf]

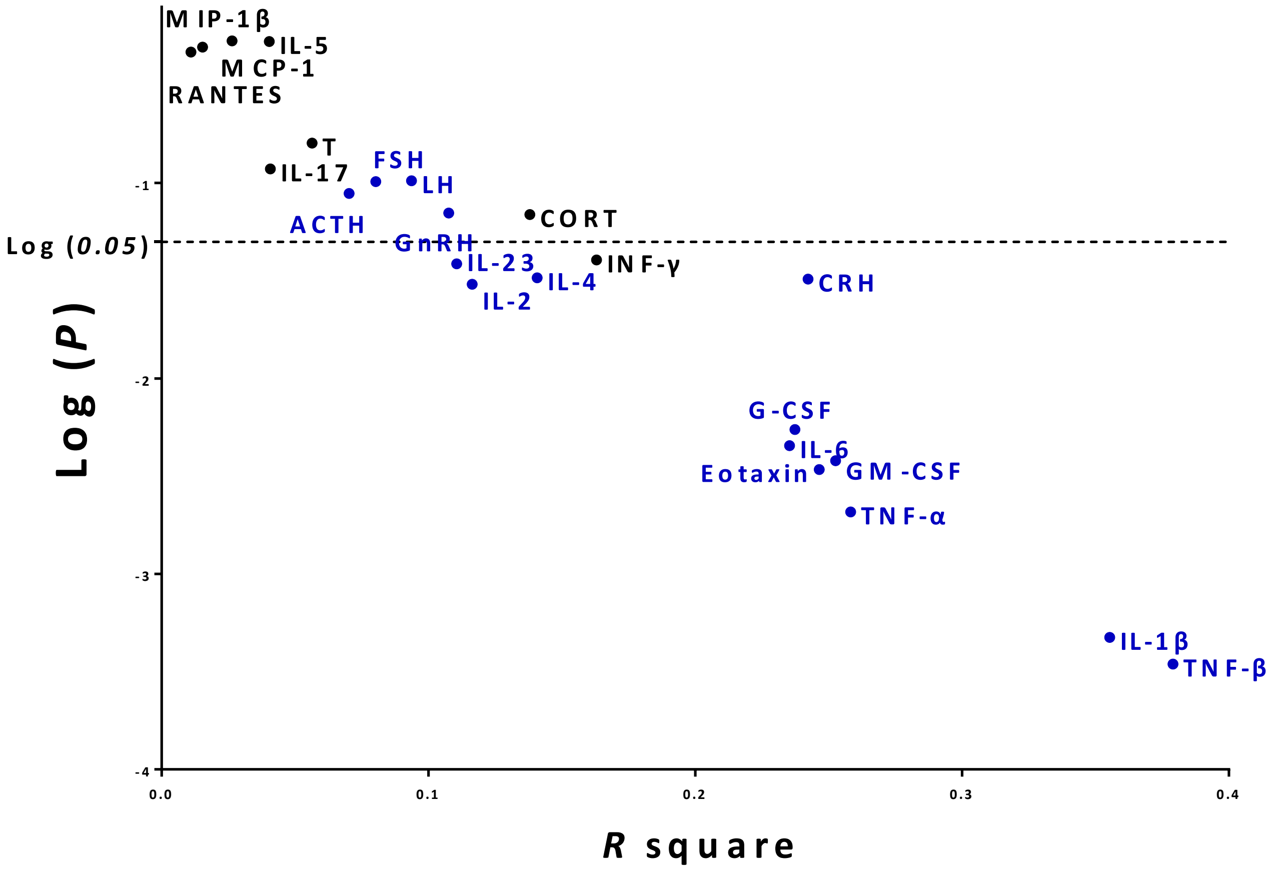

Supplemental figure 1 Correlation between endocrine hormones / cytokines and cognitive performance / pathology index of APP/PS1 mice. X and Y axis was derived by R^2 and Log (*P*), respectively. Each point represented one of endocrine hormones or cytokines in APP/PS1 mice, the blue point represented the endocrine hormone or cytokine which could be ameliorated by LW-AFC administration.
